# Oral varespladib for the treatment of snakebite envenoming in India and the USA (BRAVO): a phase II randomised clinical trial

**DOI:** 10.1136/bmjgh-2024-015985

**Published:** 2024-10-22

**Authors:** Charles J Gerardo, Rebecca W Carter, Surendra Kumar, Farshad M Shirazi, Suneetha D Kotehal, Peter D Akpunonu, Ashish Bhalla, Richard B Schwartz, Chanaveerappa Bammigatti, Neeraj Manikath, Partha P Mukherjee, Thomas C Arnold, Brian J Wolk, Sophia S Sheikh, Dawn R Sollee, David J Vearrier, Samuel J Francis, Adiel Aizenberg, Harish Kumar, Madhu K Ravikumar, Sujoy Sarkar, Taylor Haston, Andrew Micciche, Suraj C Oomman, Jeffery L Owen, Brandi A Ritter, Stephen P Samuel, Matthew R Lewin, Timothy F Platts-Mills

**Affiliations:** 1 Duke University School of Medicine, Durham, North Carolina, USA; 2 Ophirex, Inc, Corte Madera, California, USA; 3 Sardar Patel Medical College, Bikaner, Rajasthan, India; 4 University of Arizona Medical Center, University Campus, Tucson, Arizona, USA; 5 Mysore Medical College and Research Institute, Mysore, Karnataka, India; 6 University of Kentucky College of Medicine, Lexington, Kentucky, USA; 7 Post Graduate Institute of Medical Education and Research, Chandigarh, Chandigarh, India; 8 Augusta University Medical College of Georgia, Augusta, Georgia, USA; 9 Jawaharlal Institute of Postgraduate Medical Education, Puducherry, Tamil Nadu, India; 10 Government Medical College Kozhikode, Kozhikode, Kerala, India; 11 Calcutta National Medical College and Hospital, Kolkata, West Bengal, India; 12 Louisiana State University Health Sciences Center Shreveport, Shreveport, Louisiana, USA; 13 Loma Linda University Medical Center, Loma Linda, California, USA; 14 University of Florida Health Science Center Jacksonville, Jacksonville, Florida, USA; 15 University of Florida College of Medicine, Jacksonville, Jacksonville, Florida, USA; 16 The University of Mississippi Medical Center, Jackson, Mississippi, USA

**Keywords:** Snake bite, stings and other evenoming, Poisoning, Neurology

## Abstract

**Introduction:**

Snakebite envenoming (SBE) results in over 500 000 deaths or disabling injuries annually. Varespladib methyl, an oral inhibitor of secretory phospholipase A2, is a nearly ubiquitous component of snake venoms. We conducted a phase II clinical trial to assess efficacy and safety of oral varespladib methyl in patients bitten by venomous snakes.

**Methods:**

This double-blind, randomised, placebo-controlled trial enrolled patients in emergency departments in India and the USA. Patients with SBE were randomly assigned (1:1) to receive varespladib methyl or placebo two times per day for 1 week. All patients received standard of care, including antivenom. The primary outcome was change in the composite Snakebite Severity Score (SSS) measuring the severity of envenoming, from baseline to the average composite SSS at 6 and 9 hours.

**Results:**

Among 95 patients randomised August 2021 through November 2022, the most common snakebites were from Russell’s vipers (n=29), copperheads (n=18) and rattlesnakes (n=14). The SSS improved from baseline to the average at 6 and 9 hours by 1.1 (95% CI, 0.7 to 1.6) in the varespladib group versus 1.5 (95% CI, 1.0 to 2.0) in the placebo group (difference −0.4, 95% CI, −0.8 to 0.1, p=0.13). While key secondary outcomes were not statistically different by treatment group, benefit was seen in the prespecified subgroup initiating study drug within 5 hours of bite (n=37). For this early treatment group, clinically important differences were observed for illness severity over the first week, patient-reported function on days 3 and 7 and complete recovery. No death or treatment emergent serious adverse event occurred.

**Conclusion:**

For emergency department treatment of snakebites, the addition of varespladib to antivenom did not find evidence of difference for the primary outcome based on the SSS. A potentially promising signal of benefit was observed in patients initiating treatment within 5 hours of snakebite.

**WHAT IS ALREADY KNOWN ON THIS TOPIC**

**WHAT THIS STUDY ADDS**

- In this study of hospitalised patients receiving concurrent standard of care therapy including antivenom, a signal of benefit from varespladib was seen for the subgroup of patients receiving study drug within 5 hours of bite or symptom onset.
- Oral varespladib appears to be safe and well tolerated for the treatment of SBE.

**HOW THIS STUDY MIGHT AFFECT RESEARCH, PRACTICE OR POLICY**

- The results of this study are important because of the potential of oral varespladib methyl to serve as an early therapy to reduce the time from bite to treatment for this neglected tropical disease.
- This study provides the first human data regarding the potential of direct toxin inhibitors to reduce the global problem of SBE.

## Introduction

 Worldwide, snakebite envenoming (SBE) annually results in approximately 150 000 deaths and 400 000 cases of permanent disability.^1^ India has the highest country-level burden of SBE, with an estimated 58 000 annual deaths.^2^ Death and disability following SBE are also common in Southeast Asia, Africa, the Middle East and Central and South America. In the USA, an estimated 11 000 people are bitten by venomous snakes each year, resulting in both short-term and long-term disability.^3^

Antivenoms are antibody-based therapies that remain the only pharmacologic treatment for SBE. Antivenom efficacy is time-dependent, and their major limitation is that administration is often delayed. Early administration of antivenoms is often not possible because their use is generally limited to hospital settings, a result of antivenoms being intravenously administered and having the potential for serious allergic reactions. The absence of an effective and widely available field or prehospital therapy has profound consequences for victims of snakebite. In India, over 75% of deaths occur prior to hospital arrival.^4^ In sub-Saharan Africa, each hour delay in treatment is estimated to increase mortality by 1%.^5^ Additionally, the cost of antivenoms limit their supply and distribution creating further challenges to timely access to treatment in some regions.^6^ Reflecting these and other concerns, in 2017 the WHO identified snakebite as a Neglected Tropical Disease, and in 2019 the WHO Snakebite Envenoming Working Group encouraged development of oral direct toxin inhibitors, including varespladib, that can be administered in the field to address the limitation of antivenom and reduce morbidity and mortality.^7^

Secretory phospholipase A2s (sPLA2s) are the most functionally diverse and toxic of the four common snake venom components that include three-finger toxins, metalloproteases and serine proteases.^8 9^ Snake venom sPLA2s are a family of enzymes found in varying proportions in the venom of at least 95% of venomous snakes, including all venomous snakes native to the USA and India. sPLA2s act through hydrolysis of phospholipids with release of inflammatory mediators as well as through a cytotoxic pore-forming mechanism.^10^,^12^ Organ-specific injury from these toxins occurs via binding of the sPLA2 to proteins on the surface of target cells.^12 13^ Through these mechanisms, sPLA2s contribute to all major categories of SBE pathology: cytotoxicity, coagulopathy, haemolysis, nephrotoxicity, cardiovascular instability and neurotoxicity.^8^ Additionally, because of their small size, which allows for rapid diffusion and systemic effects, and their effects on neuromuscular and neurovascular function, sPLA2s have been implicated in lethal effects of SBE.^14^,^16^ The wide occurrence, abundance and severity of pathology resulting from sPLA2s make this toxin family a promising therapeutic target.

Varespladib methyl (*syn* S3013, LY333013) is an orally bioavailable small-molecule prodrug that, when de-esterified, becomes varespladib (LY351920) a potent inhibitor of snake venom sPLA2. In preclinical studies, varespladib, and its oral prodrug, varespladib methyl, have been found to rescue animals from otherwise lethal doses of viper and elapid venoms.^17 18^ If efficacious, oral varespladib could address the need for timely access to treatment for SBE by serving as an easy to administer field or prehospital treatment.

The purpose of this trial was to assess the efficacy, safety and tolerability of varespladib methyl when given in addition to concurrent standard of care, including antivenom, for patients with SBE.

## Methods

### Study design and participants

We conducted this multicentre randomised, double-blind, placebo-controlled trial at six sites in India and six sites in the USA (online supplemental appendix). The trial protocol was approved by Indian and US regulatory authorities and the Institutional Review Boards (IRB) or Ethics Committees (EC) at each site.

An independent data and safety monitoring board oversaw and periodically reviewed safety data from the trial. None of the funding agencies controlled the trial design or conduct, analysis of the data, or writing of the manuscript.

All patients received institutional standard of care, including antivenom as indicated, without any delays or restrictions. Patients 5 years of age or older who presented to Accident and Emergency with signs and symptoms of SBE were screened for eligibility. Patients were eligible if they were randomised within 10 hours of bite or symptom onset and had a Snakebite Severity Score (SSS) of 3 or more based on the composite of the local wound, pulmonary, cardiovascular, haematologic and nervous system subscores (online supplemental appendix), either from a single subscore of at least 3 or a subscore of at least 2 in one category and at least 1 in another category.^19^ The diagnosis of SBE was ascertained by site investigators, each of whom has extensive formal training and clinical experience caring for such patients. Consistent with the original SSS, the nervous system subscore was based on both neuromuscular weakness as well as manifestations of central nervous system pathology. An abnormal 20 min whole blood clotting test (20WBCT) could be used as the basis for inclusion in the trial, but the scoring of the SSS for baseline and follow-up outcomes was based on standard laboratory values, not the 20WBCT. Patients were excluded if they had a history of cerebrovascular accident, acute coronary syndrome, chronic kidney disease or chronic liver disease. There was no exclusion based on the species of the offending snake or inability to identify the snake species involved. See the online supplemental appendix and the manuscript describing the study protocol^20^ for a full list of exclusion criteria.

### Randomisation and masking

Patients were randomised in a 1:1 ratio to varespladib or placebo with concealed allocation prior to randomisation. Randomisation was blocked and stratified by age (three groups: ages 5–10, 11–17 or ≥18 years) and the presence of neurotoxicity at baseline (two groups: SSS neurologic subscore of 0–1 or 2–3). Neurotoxicity is of particular interest because it can be life-threatening and for some snake species, may be resistant to antivenom.^21^ Although not specified as a stratification variable in the statistical analysis plan, randomisation was also stratified by site. A computer-generated list was used for randomisation. The treating team, trial management group and patients remained unaware of the group assignments until the final analysis of the study was complete.

### Intervention

Adult patients randomised to the active drug received 500 mg of varespladib methyl followed by 250 mg two times per day for 7 days, administered as 250 mg tablets. The dosing for paediatric patients was 200 mg followed by 100 mg for patients aged 5–10 years and 400 mg followed by 200 mg for patients aged 11–17 years, administered as 50 mg capsules. Patients randomised to placebo received an equivalent number of placebo tablets or capsules. Both varespladib methyl and placebo tablets and capsules were made by the same manufacturer under Good Manufacturing Practices conditions. All patients also received institutional standard of care, including antivenom, at the discretion of the treating physician.

### Outcomes

The primary efficacy outcome was the change in a five-component version of the SSS (pulmonary, cardiovascular, haematologic, nervous and renal system subscores) from baseline to the average at 6 and 9 hours. This outcome differs from the original SSS as defined by Dart *et al* SSS (pulmonary, cardiovascular, haematologic, nervous, local wound and gastrointestinal system subscores) because it removes the local wound subscore, which was not expected to change over this time frame but is included in other outcomes described below, and the gastrointestinal subscore, which was deemed to be of lower clinical relevance than other subscores, and adds a renal subscore, producing a composite score with a minimum value of 0 and a maximum value of 16 (online supplemental appendix). The renal subscore was based on the Kidney Disease Improving Global Outcomes measure for acute kidney injury.^22^

Four alpha-protected key secondary efficacy outcomes were identified for sequential testing in the following order: (1) area under the curve (AUC) for the six-component SSS (local wound, pulmonary, cardiovascular, haematologic, nervous and renal system subscores; SSS AUC) from baseline to day 7; (2) amount of antivenom administered after initiation of study drug, categorised into three groups (low, medium or high; definitions provided in online supplemental appendix); (3) pain AUC from baseline to day 3, using the 0–10 numeric pain rating scale and (4) Clinician Global Impression-Improvement at day 2, using a 7-point scale. The SSS AUC was calculated by summing the area of trapezoids formed by elapsed time between assessments on the X-axis and SSS at each assessment on the Y-axis. The SSS AUC day 7 has a theoretical range from 0 to 3360, with values representing the burden of illness over the first 7 days. Calculation of the SSS AUC from baseline to day 7 was based on assessments at baseline, 3 hours, 6 hours, 9 hours, day 2, day 3 and day 7. Additional outcomes included the Patient-Specific Functional Scale (PSFS), complete recovery defined by an SSS of 0 (a post-hoc analysis) and individual subscores of the SSS.

Safety was assessed with vital signs, physical examination, laboratory tests, ECGs, clinician assessments and patient-reported symptoms and categorised by system organ class and preferred term using the Medical Dictionary for Regulatory Activities. Concomitant medication and therapies, interventions, duration of hospitalisation and adverse events were recorded. Patient input informed the selection and timing of outcome assessments in the trial.

### Statistical analysis

We determined that the enrolment of 47 patients per group (94 total) would provide the trial with 85% power to show a between-group difference in the primary outcome of 1.1 points assuming a SD of 1.75 (two-sided alpha-level 0.05). The difference of 1.1 was derived from a model of expected outcomes based on the natural history of SBE and studies of varespladib in animal models. The minimum clinically important difference for this outcome has not been defined, but a 1-point difference in the original six-component SSS is considered clinically significant.^19^

The primary analysis was performed in the intention-to-treat (ITT) population, which included all randomised patients. Missing data were imputed (details provided in the online supplemental appendix). Analysis of covariance was used to compare differences in treatment groups for the primary efficacy outcome. The a priori specified model used for the primary and key secondary analyses included adjustment for baseline illness severity measured using the SSS, age group (age 5–17 years vs age 18 and older), the absence or presence of moderate or severe neurotoxicity (baseline nervous system subscore of 0 or 1 vs 2 or 3) and country (India vs USA). Based on a review of covariates, post-hoc analyses were only adjusted for baseline SSS. Preplanned subgroup analyses included estimates of efficacy by country, snake type (viper, elapid or unknown), age group (paediatric or adult), time from bite or symptom onset to initiation of study drug (<5 hours vs ≥5 hours) and whether the patient had completed an initial dose of antivenom prior to randomisation. The amount of antivenom given to patients in each treatment arm was compared using a proportional odds logistic regression model. Adverse events were evaluated in the safety population and compared between treatment groups.

Post-hoc analyses were conducted to better characterise the effect of varespladib versus placebo on outcomes extending beyond 7 days, both for the overall population and for the subgroup of patients receiving treatment within 5 hours of bite. These analyses examined the change in the PSFS to day 3 and day 7 and complete recovery as defined by a six-component SSS of zero at each assessment through day 28. The change in PSFS from ‘baseline’ to day 3 and to day 7 was used by calculating the change from the first assessment of PSFS, which occurred at the 9 hour assessment time point. Complete recovery analyses were conducted using the ITT population with fractional imputed values rounded to 0 or 1 using usual rounding rules.

All analyses were performed with SAS V.9.4. The trial was registered at ClinicalTrials.gov, NCT04996264, and Clinical Trials Registry-India, CTRI/2021/11/037901.

### Patient and public involvement

Patients and the public were not directly involved in the design or reporting of the study. The study was designed and implemented by physicians who care for patients with SBE and who prioritised the clinical relevance of the study for these patients. The study included several patient-reported outcomes including the PSFS and components of the SSS.

## Results

### Study population

**Figure 1:**
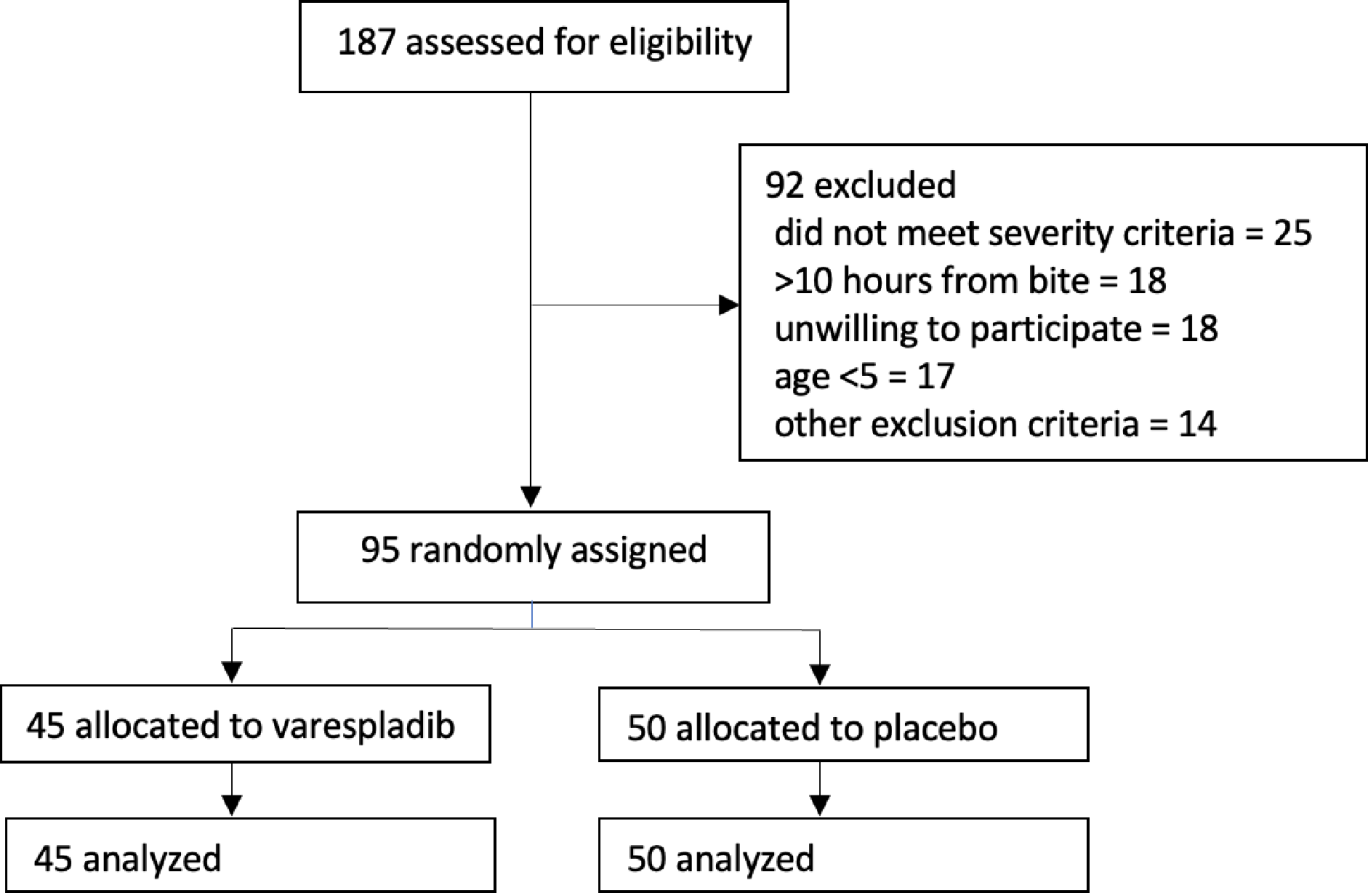
Allocation of patients in the BRAVO study.

Between 15 August 2021 and 14 November 2022, we assessed 187 potential participants for eligibility. Ninety-two people were ineligible because they did not meet inclusion criteria (n=74) or because they declined to participate (n=18; figure 1). Ninety-five patients were randomly allocated to varespladib or placebo. These 95 patients, who form the ITT population, were analysed for efficacy outcomes. One patient (randomised to placebo) consented to randomisation but declined to take the first dose of study medication or participate further in the trial. This patient is not included in the safety population. Outcome assessments were completed for 90 of 95 (95%) patients on day 2 and day 3, 87 of 95 (92%) patients at day 7 and 82 of 95 (86%) patients at day 28.

Baseline characteristics are shown in table 1. Sixty-two patients were enrolled in India; 33 patients were enrolled in the USA. Russell’s viper (*Daboia russelii*) and copperhead (*Agkistrodon contortrix*) were the most common offending snakes. Baseline mean SSS was 5.9 for the varespladib group versus 5.3 for the placebo group (difference 0.6; 95% CI, −0.25 to 1.45). The mean time from bite to treatment was 6 hours and 31 min for varespladib patients versus 5 hours and 49 min for placebo patients. The shortest time from bite to treatment was 2 hours and 42 min. At baseline, the SSS subscores contributing most to the overall score were local wound (total average of 1.9, SD=0.8), haematologic (total average of 1.6, SD=1.5) and neurologic (total average of 1.0, SD=0.8). Of the 29 patients in the study bitten by Russell’s viper, 13 had neurotoxicity, though only three had moderate or severe neurotoxicity as defined by an SSS nervous system subscore of 2 or 3. The longest duration of hospitalisation was 26 days for a 55-year-old male patient bitten by a Russell’s viper.

**Table 1:** Baseline characteristics

Characteristic	Varespladib (n=45)	Placebo (n=50)
Age—year		
Mean (SD)	38 (19)	42 (16)
5–10 years—no (%)	1 (2)	2 (4)
11–17 years—no (%)	5 (11)	2 (4)
18 years and older—no (%)	39 (87)	46 (92)
Sex		
Male—no (%)	35 (78)	39 (78)
Female—no (%)	10 (22)	11 (22)
Country		
India—no (%)	31 (69)	31 (62)
USA—no (%)	14 (31)	19 (38)
Time from bite to treatment (hours)*		
Mean (SD)	6.5 (2)	5.8 (2)
Snake type†—no (%)		
Russell’s viper	16 (36)	13 (27)
Copperhead	9 (20)	9 (18)
Rattlesnake‡	5 (11)	9 (18)
Krait	3 (7)	6 (12)
Cobra§	0	5 (10)
Saw-scaled viper	2 (4)	1 (2)
Hump-nosed viper	1 (2.2)	1 (2)
Unknown	9 (20)	6 (12)
Baseline Snakebite Severity Score		
Mean (SD)	5.9 (1.9)	5.3 (2.2)
Range	3–11	2–14

* Time from bite or symptom onset until administration of first dose of study drug.

† Krait and cobra are in the elapid family. Other identified snakes are vipers.

‡ Four suspected or confirmed species: *Crotalus atrox* (n=6), *C. horridus* (n=3), *C. willardi* (n=1), *Sistrurus miliarus* (n=1), *Crotalus spp*., species not identified (n=3).

§ *Naja naja* (n=4) and *Naja kaouthia* (n=1)

All patients in the ITT population received antivenom except for two patients enrolled in India for whom the treating physicians did not administer antivenom based on their clinical judgement. One patient (randomised to placebo) was bitten by a hump-nosed viper (*Hypnale hypnale*), a snake for which there is no specific antivenom available. The other patient (randomised to varespladib) was bitten by an unknown snake species. In the USA, patients received one of two types of antivenom: CroFab, a Fab, or ANAVIP, a F(ab’)2. In India, patients received VINS, Premium Serums or Bharat Serums antivenoms, all of which are F(ab’)2. Among the 93 patients who received antivenom, for 64 patients, antivenom was initiated prior to starting the study drug. For the remaining 29 patients, the average time from initiation of study drug to initiation of antivenom was 27 min.

### Primary and key secondary outcomes

The mean SSS change from baseline to the average at 6 and 9 hours (primary outcome adjusted for prespecified covariates) was a 1.1 improvement in the varespladib group versus a 1.5 improvement in the placebo group (difference 0.35; 95% CI, −0.8 to 0.1; table 2). In the preplanned subgroup of patients initiating study drug within 5 hours of bite, the primary outcome improved by 1.6 in the varespladib group versus 1.1 in the placebo group (difference=0.5, 95% CI, −0.3 to 1.2; table 3). A test for the interaction between treatment group and time to initiation of treatment was statistically significant (p=0.03).

**Table 2:** Primary and key secondary outcomes in the intention-to-treat population (n=95)

Outcome	Varespladib (n=45)	Placebo (n=50)	Treatment effect* (95% CI)
Primary outcome			
Change in SSS from baseline to average at 6 and 9 hours, mean (SE)	1.1 (0.3)	1.5 (0.2)	−0.4 (−0.8 to 0.1) (Favours placebo)
Key secondary outcomes			
SSS AUC day 7, mean (SE)	421 (45)	430 (44)	−9 (−91 to 73) (Favours varespladib)
Antivenom administration, low/medium/high dosing category	L=16%/M=36%/ H=49%	L=10%/M=32%/ H=56%	0.75 (0.31 to 1.79)† (Favours varespladib)
Pain Severity (0–10 scale) AUC day 3, mean (SE)	170 (17)	173 (18)	−3 (−35 to 29) (Favours varespladib)
Clinical Global Impression-Improvement day 2, mean (SE)	2.3 (0.2)	2.4 (0.2)	−0.1 (−0.5 to 0.3) (Favours varespladib)

* Estimates and treatment effects adjusted for four prespecified covariates: baseline Snakebite Severity Score, age group, baseline neurotoxicity and country.

† L, low; M, medium; H, high, in reference to the amount of antivenom administered to the patient after the initiation of the study (see online supplemental appendix for dosing categories by antivenom type). Relative odds based on a proportion model. A relative odds of 1 means no difference between varespladib and placebo. A relative odds less than 1 favours varespladib.

AUC, area under the curve; SSS, Snakebite Severity Score.

**Table 3:** Outcomes in patients treated within 5 hours of bite or symptom onset (n=37)

Outcome	Varespladib (n=17)	Placebo (n=20)	Treatment effect (95% CI)
Primary outcome*			
Change in SSS from baseline to average at 6 and 9 hours, mean (SE)	1.6 (0.6)	1.1 (0.6)	0.5 (−0.3 to 1.2) (Favours varespladib)
Key secondary outcomes*			
SSS AUC day 7, mean (SE)	263 (91)	403 (89)	−140 (−18 to −263) (Favours varespladib)
Antivenom administration, low/medium/high dosing category	12%/18%/70%	5%/20%/75%	0.78 (0.15 to 4.06) (Favours varespladib)
Pain Severity (0–10 scale) AUC day 3, mean (SE)	175 (46)	185 (50)	−10 (−75 to 55) (Favours varespladib)
Clinical Global Impression-Improvement day 2, mean (SE)	2.22 (0.2)	2.23 (0.2)	−0.01 (−0.8 to 0.7) (Favours varespladib)
Secondary outcomes†			
Change in Patient-Specific Functional Scale to day 3‡	2.99	1.56	1.43 (0.22 to 2.63) (Favours varespladib)
Change in Patient-Specific Functional Scale to day 7‡	4.62	3.74	0.88 (−0.48 to 2.24) (Favours varespladib)
Post-hoc outcomes†			
Complete recovery day 14	41%	25%	19% (−11% to 49%) (Favours varespladib)
Complete recovery day 28	61%	31%	31% (1% to 61%) (Favours varespladib)

* Estimates and treatment effects adjusted for four prespecified covariates: baseline SSS, age group, baseline neurotoxicity and country.

† Estimates and treatment effects adjusted for baseline SSS.

‡ For the Patient-Specific Functional Scale (PSFS), the change to day 3 and to day 7 is measured from the initial assessment, which occurred at 9 hours. PSFS was not assessed at baseline.

SSS, Snakebite Severity Score.

The four key secondary outcomes were also not statistically significant (table 2). In the preplanned subgroup of patients treated within 5 hours of bite, SSS AUC day 7 (ie, burden of illness over the first 7 days) was lower in the varespladib group than the placebo group (difference = −140, 95% CI, −18 to −263, nominal p value=0.02). In patients treated within 5 hours, the three other key secondary outcomes favoured varespladib but were not statistically significant (table 3). Also noted in the analysis is that among the entire ITT population, the burden of illness over the first 7 days did not differ by treatment group among subgroups defined by the baseline presence or absence of neurotoxicity or haemotoxicity.

### Other secondary and post-hoc outcomes

For all 95 patients, the improvement in the PSFS from the first assessment at 8–10 hours to day 3 was 2.2 in the varespladib group versus 1.9 in the placebo group, and the improvement from 8 to 10 hours to day 7 was 4.2 in the varespladib group and 3.7 in the placebo group. For patients initiating treatment within 5 hours of the bite, the improvement in the PSFS from 8 to 10 hours to day 3 was 3.0 in the varespladib group and 1.6 in the placebo group (nominal p value=0.02; table 3).

**Figure 2:**
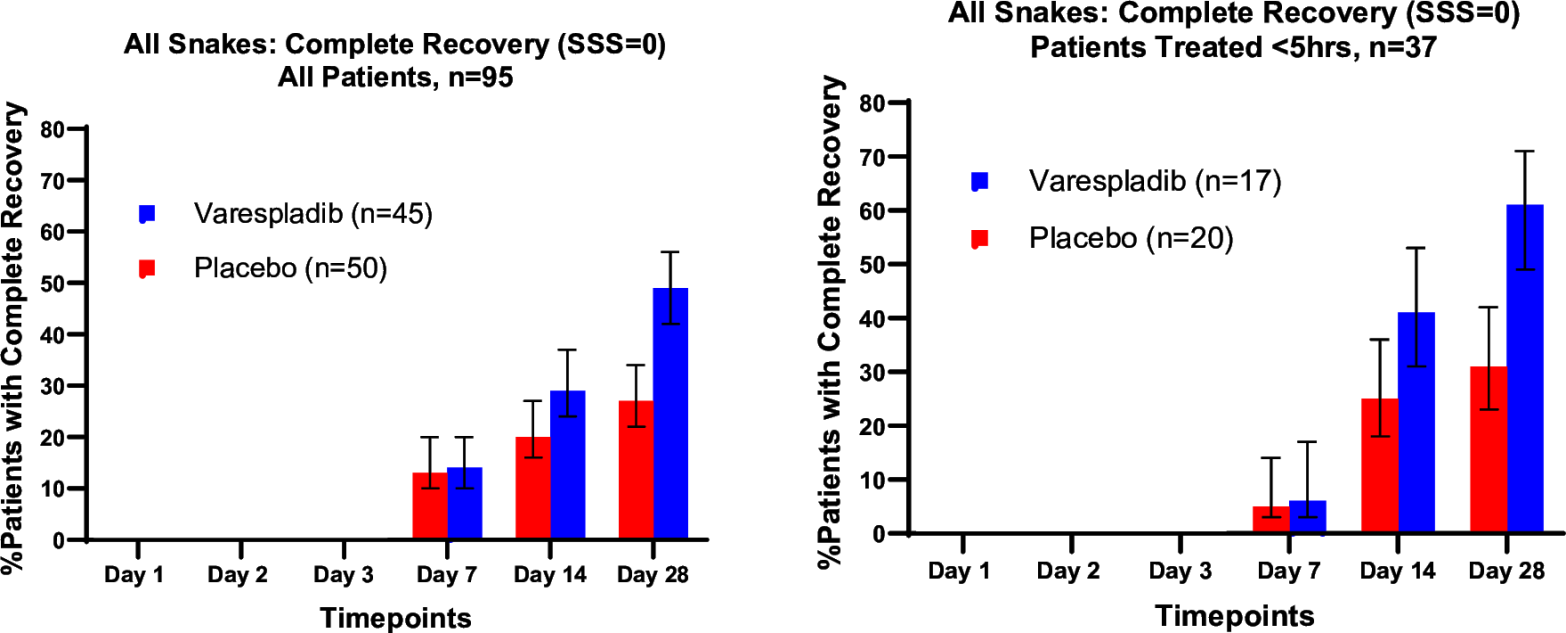
Complete recovery defined by a six-component Snakebite Severity Score (SSS) of zero, adjusted for baseline SSS. Bars indicate SE.

For all 95 patients, complete recovery (SSS=0) at day 28 occurred for 49% and 27% of the varespladib and placebo-treated patients, respectively. For patients treated within 5 hours, complete recovery at day 28 occurred for 58% and 27% of varespladib and placebo-treated patients, respectively (table 3, figure 2). Among patients treated within 5 hours, differences in complete recovery at day 28 favoured the varespladib group for patients bitten by each of the major groups of vipers represented in this study (Russell’s viper, copperhead and rattlesnakes), as well as for krait-bite patients (online supplemental appendix).

The time course for resolution of individual subscores within the SSS varied (online supplemental appendix). Neurologic subscores improved gradually over the first 7 days. Local wound subscores were unchanged through day 3, then improved from day 3 to day 14. Haematologic subscores improved gradually over the first 2 weeks. Elevated renal subscores were uncommon, but when present were persistently abnormal through at least day 7. Cardiovascular subscores improved over the first 3 to 9 hours, likely as the result of routine emergency department resuscitative practices, and were similar in both treatment arms.

### Safety outcomes

No death, no serious adverse event and no investigator request to discontinue study drug or unblind a patient occurred. There were no cases of stroke, acute coronary syndrome or malignant cardiac arrhythmia. No patient was found to have ECG changes to suggest ischaemia at any time point. The percentage of patients experiencing one or more treatment-emergent adverse events was 38% and 55% in the varespladib and placebo groups, respectively (nominal p value accounting for duration of follow-up=0.08). The frequency of selected adverse events of interest was either similar or less common in the varespladib group than in the placebo group (table 4). Among paediatrics patients aged 5–17 years, treatment-emergent adverse events occurred in 1 of 6 (17%) patients receiving varespladib versus 3 of 4 (75%) of patients receiving placebo (see details in online supplemental appendix). Varespladib was well tolerated as evidenced by low rates of nausea, vomiting, headache, fatigue or dermatologic adverse events. No patient in either treatment group was discontinued from the study drug because of an adverse event.

**Table 4:** Safety and tolerability measures

Safety outcome—no (%)	Varespladib (n=45)	Placebo (n=49)
Patients with at least one TEAE	17 (37.8%)	27 (55.1%)
Gastrointestinal disorders	4 (9%)	7 (14%)
Nausea	1 (2%)	5 (10%)
Vomiting	2 (4%)	2 (4%)
Diarrhoea	1 (2%)	1 (2%)
Infections	2 (4%)	5 (10%)
Wound infections	1 (2%)	3 (6%)
Allergic reaction to immunoglobulin	1 (2%)	3 (6%)
Acute kidney injury*	3 (7%)	2 (4%)
Hepatic enzyme increase†	1 (2%)	1 (2%)
Cardiac ischaemia	0	0
Skin/subcutaneous tissue disorders	1 (2%)	4 (8.2%)
Fatigue	0	1 (2%)
Nervous system disorders	4 (8.9%)	5 (10.2%)
Headache	2 (4.4%)	5 (10.2%)

* Defined as a creatinine either ≥0.3 mg/dL above baseline or ≥1.5 times baseline. Patients with creatinine above the upper limit of normal at baseline are excluded.

† Defined as liver enzymes, either AST or ALT, exceeding 3x the upper limit of normal. Patients with hepatic enzymes above the upper limit of normal at baseline are excluded.

ALT, alanine aminotransferase; AST, aspartate aminotransferase; TEAE, treatment-emergent adverse events.

## Discussion

In this phase II clinical trial of patients with SBE, the addition of varespladib to standard of care, including the use of antivenom did not result in a difference in the SSS measured as a change from baseline to 6 and 9 hours postbaseline (primary outcome). Key secondary outcomes slightly favoured varespladib but were not statistically significant. Importantly, however, within the prespecified subgroup of patients treated within 5 hours of the snakebite, clinically important differences and nominal statistical significance were observed for patients receiving varespladib versus placebo for the SSS AUC day 7 and for measures of physical function and recovery.

The primary results from the BRAVO study should be considered within the context of widely reproduced preclinical data and the promising additional subgroup analyses.^17 18^ Factors that may have hindered the ability to identify benefit in the primary outcome in this trial include (1) limitations of the outcome measure, (2) initiation of study drug an average of 6 hours after the bite, (3) efficacy signal obscured by concurrent administration of antivenom as well as resuscitative and symptomatic treatments, (4) random but important baseline differences in time to treatment and severity favouring the placebo group with the potential for residual confounding and (5) heterogeneity in the severity and clinical manifestations of SBE.

There is not an accepted single outcome measure for global SBE clinical trials.^23^ SBE is a heterogeneous disease with diverse clinical syndromes driven by variation in venom composition. To address this heterogeneity, a global consortium of SBE researchers currently recommend collecting a wide variety of ‘core outcome’ measures.^23^ These ‘core outcomes’ include key clinical signs, laboratory values and clinician-reported and patient-reported outcomes that evaluate important endpoints ranging from organ-specific toxicity to disability and mortality.

The SSS is an existing embodiment of the ‘core outcomes’ recommendation and addresses the challenge of diverse toxicities from SBE by using a composite measure summing subscores across multiple organ systems. To begin updating the SSS for international use, we modified it by removing the gastrointestinal subscore and adding a renal subscore which better aligns the SSS with toxicities that contribute most to morbidity and mortality globally. Nonetheless, the SSS still has limitations. Rapid death from venom neurotoxicity is generally caused by neuromuscular blockade resulting in weakness or total paralysis, but the existing neurologic subscore of the SSS focuses mostly on central nervous system effects. Objective measures of neuromuscular weakness, such as head-lift and grip strength, may better capture the most common patient conditions that put them at risk for death or reliance on advanced life-preserving measures.^24^

The timing of outcome assessments is also important. Based on findings from studies of varespladib in animal models with lethal doses of venoms, we selected a primary outcome measured 6 and 9 hours after randomisation. The results of this trial confirm that, regardless of treatment arm, some toxicities resolve within 9 hours (eg, cardiovascular) and some are unchanged over the first 48 hours (eg, local wound and blood components that do not normally regenerate in 6–9 hours). The challenge of identifying a time frame for recovery that captures benefit for different toxicities is a study design issue that will need to be addressed in future global SBE studies. A measure that integrates differences observed over time, such as AUC, may provide a practical solution to this challenge.

Patient-reported physical function during recovery from SBE is an important patient-centred aspect of SBE that has been incorporated into prior studies. More specifically, the PSFS is an extensively validated, patient-reported measure that has had an in-depth psychometric evaluation in SBE.^25^ In patients treated within 5 hours, the point estimate for the increase in PSFS at day 3 for varespladib versus placebo exceeded the established minimum clinically important difference of 1.0 on the 11-point PSFS scale. We augmented this analysis of recovery by performing a post-hoc evaluation of the proportion of patients achieving complete recovery, defined as an SSS of 0. This outcome provides an intuitive and unambiguous clinical endpoint that approximates the condition under which a patient has had resolution of symptoms. The strength of the complete recovery data is supported by the size of the treatment effect as well as the consistency of directional benefit for different snake types. One possible mechanism by which varespladib may promote recovery is by reducing the total injury experienced by the patient, thus reducing the time required for physiologic homeostasis and tissue repair.

The concurrent administration of standard of care, including antivenom, likely influenced the outcomes in this trial. A distinguishing characteristic of an oral direct toxin inhibitor is the potential for use as a field or prehospital treatment of SBE prior to antivenom administration. Studies conducted where antivenom is not available, such as in the field, community health clinics or ambulances, would be informative, but the logistics of conducting rigorous trials in these settings are daunting. Withholding antivenom when available in the hospital would be unethical without further evidence of the efficacy of varespladib alone.

Nonetheless, in this study of hospitalised patients receiving concurrent standard of care, a signal of benefit from varespladib was seen for the subgroup of patients receiving study drug within 5 hours of bite or symptom onset when evaluating multiple toxicities over time using the SSS AUC day 7 and when evaluating function and recovery. This signal is biologically plausible because SBE is a time-dependent disease for which early treatment is essential to optimise outcomes.^26 27^ Because sPLA2s are relatively small and highly active toxins that diffuse rapidly to produce systemic effects, early treatment may be particularly important when using an sPLA2 inhibitor.^8 17^ It is encouraging to see consistent benefits for recovery across the major snake types included in the study. As such, these findings are supportive of the use of varespladib as an oral treatment that can be carried in remote and austere environments and taken shortly after envenoming to minimise morbidity and mortality.

The BRAVO trial results suggest that varespladib is safe and well tolerated for the treatment of SBE. These results add to a large body of safety data from earlier trials of varespladib for other indications. Two notable safety findings were observed in this earlier work. First, a 1.2% increase in recurrent myocardial infarction was observed in a study of patients hospitalised with acute coronary syndrome who had risk factors for recurrent myocardial infarction.^28^ No increase in cardiac events was observed in two large studies of patients with stable coronary artery disease or any of the trials of varespladib for other indications.^29 30^ In the BRAVO trial, there were no cardiac events and no patients in either treatment group had ECG findings suggestive of ischaemia. Second, increases in hepatic enzymes equal to or exceeding 3× the upper limit of normal were observed in 4% (15 of 396) of patients treated with varespladib for a period of 8 weeks in two studies of stable coronary artery disease.^29 30^ All patients were asymptomatic and there was no accompanying increase in bilirubin or alkaline phosphatase. The duration of treatment tested in BRAVO is substantially shorter than these earlier trials. Furthermore, in the BRAVO trial, among patients with normal liver enzymes at baseline, no patient randomised to varespladib had an elevation in hepatic enzymes equal to or exceeding 3× the upper limit of normal through 28 days of follow-up.

Because of their small size, children are disproportionately affected by SBE.^25^ This is even more evident in low-income settings.^31^ Even as the number of paediatric patients treated in this study was small, encouraging safety data for the paediatric patients adds to a large body of safety data and should inform benefit–risk assessments about appropriate use of varespladib in children bitten by venomous snakes.

The BRAVO trial lays an important foundation for additional clinical studies of direct toxin inhibitors such as varespladib and other treatments for SBE. The signal consistent with benefit in patients treated within 5 hours of the bite is encouraging because it is biologically plausible, consistent with antivenom studies and points to the potential value of varespladib as a field or prehospital treatment. Future studies should consider focusing on patients initiating treatment within 5 or 6 hours and consider outcomes such as the SSS AUC day 7, the PSFS or measures of recovery.

This study has several limitations. Imbalances between the treatment groups included a longer time from bite to initiation of study drug and a higher baseline SSS in the varespladib group, creating the possibility of residual imbalances that may have impacted comparisons. There was no control over the timing or amount of antivenom given to patients. Long-term physical and psychological disability are important components of the total burden of SBE but were not within the scope of this study. This trial does not provide direct information regarding the value of varespladib administered without antivenom, for example, as a stand-alone or initial field therapy.

## Conclusion

Our findings support the safety of varespladib methyl for SBE from diverse snake types and when the snake type is unknown. When given in conjunction with standard of care including antivenom, the addition of varespladib did not demonstrate evidence of benefit based on the SSS at 6 and 9 hours after bite. A signal of benefit was observed for the subgroup of patients initiating treatment with varespladib within 5 hours of the bite. These results are important because of the potential of oral varespladib to serve as a field therapy to reduce the time from bite to treatment for this neglected tropical disease for which upwards of 75% of deaths occur prior to hospital arrival.

## Supplementary material

10.1136/bmjgh-2024-015985 online supplemental file 1

## Data Availability

Data are available upon reasonable request.

## References

[1] Warrell DA, Williams DJ (2023). Clinical aspects of snakebite envenoming and its treatment in low-resource settings. The Lancet.

[2] Suraweera W, Warrell D, Whitaker R (2020). Trends in snakebite deaths in India from 2000 to 2019 in a nationally representative mortality study. Elife.

[3] Jaramillo JD, Hakes NA, Tennakoon L (2019). The “T’s” of snakebite injury in the USA: fact or fiction?. *Trauma Surg Acute Care Open*.

[4] Mohapatra B, Warrell DA, Suraweera W (2011). Snakebite mortality in India: a nationally representative mortality survey. PLoS Negl Trop Dis.

[5] Habib AG, Abubakar SB (2011). Factors affecting snakebite mortality in north-eastern Nigeria. Int Health.

[6] Habib AG, Musa BM, Iliyasu G (2020). Challenges and prospects of snake antivenom supply in sub-Saharan Africa. PLoS Negl Trop Dis.

[7] Williams DJ, Faiz MA, Abela-Ridder B (2019). Strategy for a globally coordinated response to a priority neglected tropical disease: Snakebite envenoming. PLoS Negl Trop Dis.

[8] Gutiérrez JM, Lomonte B (2013). Phospholipases A2: unveiling the secrets of a functionally versatile group of snake venom toxins. Toxicon.

[9] Tasoulis T, Isbister GK (2023). A current perspective on snake venom composition and constituent protein families. Arch Toxicol.

[10] Ward RJ, Chioato L, de OLIVEIRA AHC (2002). Active-site mutagenesis of a Lys49-phospholipase A2: biological and membrane-disrupting activities in the absence of catalysis. Biochem J.

[11] Lomonte B, Moreno E, Tarkowski A (1994). Neutralizing interaction between heparins and myotoxin II, a lysine 49 phospholipase A2 from Bothrops asper snake venom. Identification of a heparin-binding and cytolytic toxin region by the use of synthetic peptides and molecular modeling. J Biol Chem.

[12] Kini RM, Evans HJ (1989). A model to explain the pharmacological effects of snake venom phospholipases A2. Toxicon.

[13] Lambeau G, Schmid-Alliana A, Lazdunski M (1990). Identification and purification of a very high affinity binding protein for toxic phospholipases A2 in skeletal muscle. J Biol Chem.

[14] Nisenbom HE, Perazzo JC, Monserrat AJ (1986). Contribution of phospholipase A2 to the lethal potency of Bothrops alternatus (víbora de la cruz) venom. Toxicon.

[15] Gutiérrez JM, Lewin MR, Williams DavidJ (2020). Varespladib (LY315920) and Methyl Varespladib (LY333013) Abrogate or Delay Lethality Induced by Presynaptically Acting Neurotoxic Snake Venoms. Toxins (Basel).

[16] Vuong NT, Jackson TNW, Wright CE (2021). Role of Phospholipases A2 in Vascular Relaxation and Sympatholytic Effects of Five Australian Brown Snake, Pseudonaja spp., Venoms in Rat Isolated Tissues. Front Pharmacol.

[17] Lewin M, Samuel S, Merkel J (2016). Varespladib (LY315920) Appears to Be a Potent, Broad-Spectrum, Inhibitor of Snake Venom Phospholipase A2 and a Possible Pre-Referral Treatment for Envenomation. Toxins (Basel).

[18] Lewin MR, Carter RW, Matteo IA (2022). Varespladib in the Treatment of Snakebite Envenoming: Development History and Preclinical Evidence Supporting Advancement to Clinical Trials in Patients Bitten by Venomous Snakes. Toxins (Basel).

[19] Dart RC, Hurlbut KM, Garcia R (1996). Validation of a severity score for the assessment of crotalid snakebite. Ann Emerg Med.

[20] Carter RW, Gerardo CJ, Samuel SP (2023). The BRAVO Clinical Study Protocol: Oral Varespladib for Inhibition of Secretory Phospholipase A2 in the Treatment of Snakebite Envenoming. Toxins (Basel).

[21] Prasarnpun S, Walsh J, Awad SS (2005). Envenoming bites by kraits: the biological basis of treatment-resistant neuromuscular paralysis. Brain (Bacau).

[22] Kellum JA, Lameire N, Aspelin P (2012). Kidney disease: Improving global outcomes (KDIGO) acute kidney injury work group. KDIGO clinical practice guideline for acute kidney injury work group. KDIGO clinical practice guideline for acute kidney injury. Kidney Int.

[23] Abouyannis M, Esmail H, Hamaluba M (2023). A global core outcome measurement set for snakebite clinical trials. Lancet Glob Health.

[24] Bickler PE, Abouyannis M, Bhalla A (2023). Neuromuscular Weakness and Paralysis Produced by Snakebite Envenoming: Mechanisms and Proposed Standards for Clinical Assessment. Toxins (Basel).

[25] Gerardo CJ, Vissoci JRN, de Oliveira LP (2019). The validity, reliability and minimal clinically important difference of the patient specific functional scale in snake envenomation. PLoS One.

[26] Gerardo CJ, Vissoci JRN, Evans CS (2019). Does This Patient Have a Severe Snake Envenomation?. JAMA Surg.

[27] Gopalakrishnan M, Saurabh S, Sagar P (2022). A simple mortality risk prediction score for viper envenoming in India (VENOMS): A model development and validation study. PLoS Negl Trop Dis.

[28] Nicholls SJ, Kastelein JJP, Schwartz GG (2014). Varespladib and cardiovascular events in patients with an acute coronary syndrome: the VISTA-16 randomized clinical trial. JAMA.

[29] Rosenson RS, Elliott M, Stasiv Y (2011). Randomized trial of an inhibitor of secretory phospholipase A2 on atherogenic lipoprotein subclasses in statin-treated patients with coronary heart disease. Eur Heart J.

[30] Rosenson RS, Hislop C, Elliott M (2010). Effects of varespladib methyl on biomarkers and major cardiovascular events in acute coronary syndrome patients. J Am Coll Cardiol.

[31] Bhaumik S, Menon GR, Habib AG (2023). Prioritising snakebite in the child and adolescent health agenda. Lancet Child Adolesc Health.

