# Supplementary material for: Oral varespladib for the treatment of snakebite envenoming in India and the USA (BRAVO): a phase II randomised clinical trial
Source: BMJ Glob Health. 2024 Oct 22;9(10):e015985. doi: 10.1136/bmjgh-2024-015985 (PMC11499837; doi:10.1136/bmjgh-2024-015985)
Supplement: online supplemental file 1 [file bmjgh-9-10-s001.pdf]

Supplementary Appendix

Appendix 1: Location and list of sites in India and the U.S.

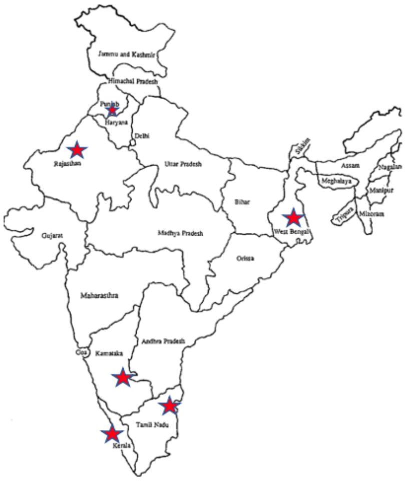

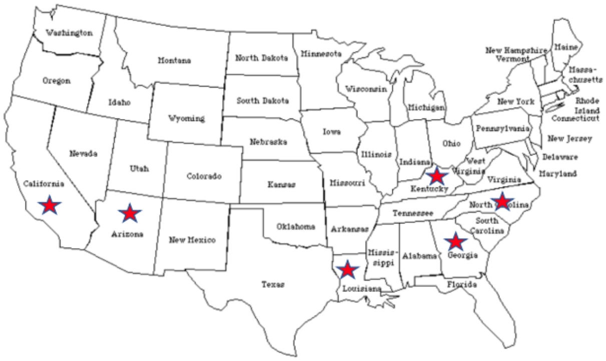

<u>India</u>

1. S. P. Medical College, Bikaner, Rajasthan
2. Post Graduate Institute of Medical Education & Research (PGIMER), Chandigarh
3. Calcutta National Medical College & Hospital, Kolkata, West Bengal
4. Jawaharlal Institute of Postgraduate Medical Education & Research (JIPMER), Puducherry
5. Government Medical College, Kozhikode, Kerala
6. K. R. Hospital, Mysore Medical College & Research Insititute, Mysore, Karnataka

<u>United States</u>

7. Duke University, Durham, North Carolina
8. University of Kentucky, Lexington, Kentucky
9. Loma Linda University, Loma Linda, California
10. University of Arizona, Tucson, Arizona
11. Augusta University, Augusta, Georgia
12. Ochsner LSU Health, Shreveport, Louisiana

Appendix 2: Complete Eligibility Criteria.

<u>Inclusion Criteria</u>

1. Is a male or female ≥ 5 years of age with venomous snakebite.
2. Index event (snakebite) must be symptomatic and symptom onset must have occurred within 10 hours of eligibility assessment.
3. Patients must meet one of two categories of inclusion criteria:
    - Category 1: The patient has not yet completed first dose of antivenom: SSS inclusion score* of ≥2 in one system and ≥1 in another system (2+1) OR ≥3 in at least one system. OR
    - Category 2: The patient has completed an initial dose of antivenom: SSS inclusion score* of ≥2 in one system and ≥1 in another system (2+1) OR ≥3 in at least one system AND CGI-I score of ≥5 (i.e., minimally worse, much worse, or very much worse).
4. Is willing (or legally authorized representative is willing) to provide informed consent prior to initiation of any study procedures.

*Only local wound, pulmonary, cardiovascular, haematologic, or nervous system scores qualify for SSS inclusion criteria. GI and Renal scores are not used for inclusion. Haematologic score may be counted if available, but inclusion should not wait for laboratory results. Point of care tests (e.g., 20WBCT) may be used for enrollment, if used per site standard of care.

<u>Exclusion Criteria</u>

1. Is considered by the Investigator to have a clinically significant upper GI bleed evidenced by hematemesis, "coffee-ground" emesis or nasogastric aspirate, or hematochezia thought to originate from upper GI tract.
2. Has history of cerebrovascular accident or intracranial bleeding of any kind, acute coronary syndrome, myocardial infarction, or severe pulmonary hypertension.
3. Has known history of inherited bleeding or coagulation disorder.
4. Is, at Screening Visit, using the following anticoagulants: warfarin/coumadin, argatroban, bilvalirudin, lepirudin, apixaban, dabigatran, clopidogrel, prasugrel, ticlodipine or another anticoagulant agent not specifically listed, or has used heparin, enoxaparin, fondaparinux, or other low molecular weight heparin or antiarrhythmic drugs within 14 days prior to treatment.
5. Has a history of chronic liver disease such as chronic active viral hepatitis, alcohol-related liver disease, non-alcoholic steatohepatitis, non-alcoholic fatty liver disease, haemochromatosis, primary biliary cirrhosis, primary sclerosing cholangitis, autoimmune hepatitis.
6. Reports or has known pre-existing renal impairment or chronic kidney disease.
7. Has a known allergy or significant adverse reaction to varespladib-methyl.
8. Is considered by the Investigator to be unable to comply with protocol requirements due to geographic considerations, psychiatric disorders, or other compliance concerns.
9. Is pregnant, has a positive urine or serum human chorionic gonadotropin (hCG) pregnancy test or not willing to use a highly effective method of contraception for 14 days after initial treatment, or is breast-feeding.

Appendix 3: Snakebite Severity Score versions used for (i) Inclusion Criteria; (ii) Primary Outcome; (iii) Secondary Outcomes (SSS AUC basline to Day 7 and complete recovery).

**Local wound #,^**	
No signs/symptoms	0
Pain, swelling, or ecchymosis within 5–7.5 cm of bite site	1
Pain, swelling, or ecchymosis involving less than half the extremity (7.5–50 cm from bite site)	2
Pain, swelling, or ecchymosis involving half to all of extremity (50–100 cm from bite site)	3
Pain, swelling, or ecchymosis extending beyond affected extremity (more than 100 cm of bite site)	4
**Pulmonary system #,*,^**	
No signs/symptoms	0
Dyspnea, minimal chest tightness, mild/vague discomfort, respirations of 20–25 breaths per minute	1
Moderate respiratory distress, 26–40 bpm	2
Cyanosis, air hunger, extreme tachypnea, or respiratory insufficiency /failure	3
**Cardiovascular system #,*,^**	
No signs/symptoms	0
HR 100–125 BPM, palpitations, generalized weakness, benign dysrhythmia, or hypertension	1
HR 126–175 BPM, or hypotension with SBP > 100 mmHg	2
HR > 175 BPM, or hypotension with SBP < l00 mmHg, malignant dysrhythmia, or cardiac arrest	3
**Gastrointestinal system**	
No signs/symptoms	0
Pain, tenesmus, or nausea	1
Vomiting or diarrhea	2
Repeated vomiting, diarrhea, hematemesis, or hematochezia	3
**Haematologic system #,*,^**	
No signs/symptoms	0
Coagulation parameters slightly abnormal: PT ULN–20 secs, PTT ULN–50 secs, platelets 100–150K/mL, or fibrinogen 100–150 mcg/mL	1
Coagulation parameters abnormal: PT 20–50 secs, PTT 50–75 secs, platelets 50–100K/mL, or fibrinogen 50–100 mcg/mL	2
Coagulation parameters abnormal: PT 50–100 secs, PTT 75–100 secs, platelets 20–50K/mL, or fibrinogen < 50 mcg/mL	3
Coagulation parameters markedly abnormal, with serious bleeding or the threat of spontaneous bleeding; unmeasurable PT or PTT, platelets < 20 K/mL, undetectable fibrinogen, severe abnormalities of other laboratory values also fall into this category	4
**Nervous system #,*,^**	
No signs/symptoms	0
Minimal apprehension, headache, weakness, dizziness, chills, or paresthesia	1
Moderate apprehension, headache, weakness, dizziness, chills, paresthesia, confusion, fasciculation in area of bite site, ptosis, or dysphagia	2
Severe confusion, lethargy, weakness, paralysis, seizures, coma, psychosis, or generalized fasciculation	3
**Renal system*,^**	
Normal creatinine and urine output	0
Creatinine 1.5 to 1.9 times baseline, increase in creatinine ≥0.3mg/dl (≥26.5 μmol/L) from baseline, or urine output <0.5 ml/kg/h for >6 h	1
Creatinine 2 to 2.9 times baseline or urine output <0.5 ml/kg/h for >12 h	2
Creatinine ≥3.0 times baseline, increase in creatinine to ≥4.0 mg/dl (≥353.6 μmol/L), urine output <0.3 ml/kg/h for ≥24 h or anuria ≥12 h, or initiation of renal replacement therapy	3
**TOTAL**	

# Subscores used for inclusion determination. Haematologic subscore may be used if available, but enrollment should not be delayed. Abnormal 20WBCT may be used for inclusion.
* Subscores used for primary endpoint. Definitive laboratory testing should be used for efficacy endpoints.
^ Subscores used for secondary Outcomes (SSS AUC baseline to Day 7 and complete recovery)

Appendix 4: Imputation methods.
The multiple imputation (MI) procedures for the primary and key secondary endpoints are described below. For the primary analysis, missing data not due to mortality, and not addressed by the averaging rules above, will be imputed using the following regression-based multiple imputation model. The following imputation algorithm will be applied to each SSS subscore separately, then the primary outcome SSS score will be derived.

1. The first step is to understand the pattern of missingness. In order to perform the multiple imputation, a monotone missing pattern must be achieved. For example, if there exist values for baseline and Day 2 visits, but missing values for the Day 1 (8-10h) visit, Markov-Chain Monte-Carlo (MCMC) method will be used to impute the small amount of missing data that may be missing at the intermediate visits that is required to make the missing data pattern monotone before applying the multiple imputation algorithm. This method uses a non-informative Jeffreys prior to derive the posterior mode from the expectation-maximization (EM) algorithm as the starting values for the MCMC method. This MCMC method will use seed of 432832. To avoid values that could not be observed in practice, imputed values will be constrained to be integers in the range of 0 to 3 or 0 to 4 by convention to align with the scale of the questionnaire. A total of 10 imputations will be done in the MCMC step.
2. Once the monotone pattern is achieved, the next step is to implement the imputation algorithm. For this, the Predictive Mean Matching method (PMM) will be used. This method is particularly helpful if the normality assumption is violated. For patients with complete data up to a particular visit, a PMM model will be fit that includes the outcome at that visit as the dependent variable; and as independent variables gender, treatment assignment, study site, and non-missing scores using a seed of 931428. This process will be repeated 15 times, resulting in a total of 150 complete analysis datasets.
3. For each completed dataset, any necessary derived variables will be computed. Then the ANCOVA model will be performed for the 6 and 9 hour average SSS score. The results will be combined into one MI inference (LS mean, associated 95% CI, and p-value) using PROC MIANALZE as illustrated.(Ratitch et al., 2013)

The first key secondary analysis on AUC will use the same process above, except in step 3 the AUC will be derived for each patient in each imputed dataset, then analysed and aggregated as above using PROC MIANALZE.(Ratitch et al., 2013) The secondary key secondary analysis on antivenom amounts will not use multiple imputation. The third key secondary analysis on NPRS AUC will use the same process above, except in step 3 the NPRS-AUC will be derived for each patient in each imputed dataset, then analysed and aggregated as above using PROC MIANALZE.(Ratitch et al., 2013) The fourth key secondary analysis on CGI-I will use the same MI method described above, except using baseline and post-baseline CGI-I scores.

Ratitch, B., O'Kelly, M., & Tosiello, R. (2013). Missing data in clinical trials: From clinical assumptions to statistical analysis using pattern mixture models. *Pharmaceutical Statistics*, *12*(6). https://doi.org/10.1002/pst.1549

Appendix 5: Outcomes in patients treated ≥5 hours of bite or symptom onset (n=58).

Outcome	Varespladib (n=28)	Placebo (n=30)	Treatment Effect (95% CI)
*Primary outcome**			
Change in SSS from baseline to average at 6 and 9 hours, mean (SE)	1.0 (0.3)	1.7 (0.3)	-0.7 (-1.3 to -0.1)
*Secondary outcomes**			
SSS AUC to Day 7, mean (SE)	473 (55)	428 (54)	45 (-65 to 155)
Antivenom administration, low/medium/high dosing category	18% / 46% / 36%	14% / 41% / 45%	0.58 (0.19 to 1.72)
Pain Severity (0-10 scale) AUC Day 3, mean (SE)	166 (18)	164 (18)	2 (-34 to 39)
Clinical Global Impression-Improvement Day 2, mean (SE)	2.38 (0.3)	2.24 (0.2)	0.13 (-0.35 to 0.61)
*Post-hoc outcomes***			
Change in Patient Specific Functional Scale to Day 3***	1.79	2.26	-0.47 (-1.73 to 0.78)
Change in Patient Specific Functional Scale Day 7***	3.76	3.69	0.07 (-1.39 to 1.52)
Complete recovery Day 14	22%	19%	3% (-17% to 23%)
Complete recovery Day 28	41%	27%	14% (-10% to 38%)

*Estimates and treatment effects adjusted for four pre-specified covariates: baseline SSS, age group, baseline neurotoxicity, and country.
**Estimates and treatment effects adjusted for baseline SSS.
*** For the Patient Specific Functional Scale (PSFS), the change to Day 3 and to Day 7 is measured from the initial assessment, which occurred at 9 hours. PSFS was not assessed at baseline.

Appendix 6: Complete recovery by treatment group for patients bitten by the four most common snake types in the trial. Error bars show standard error.

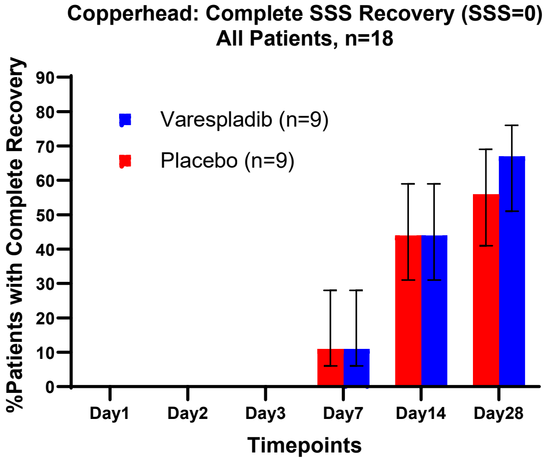

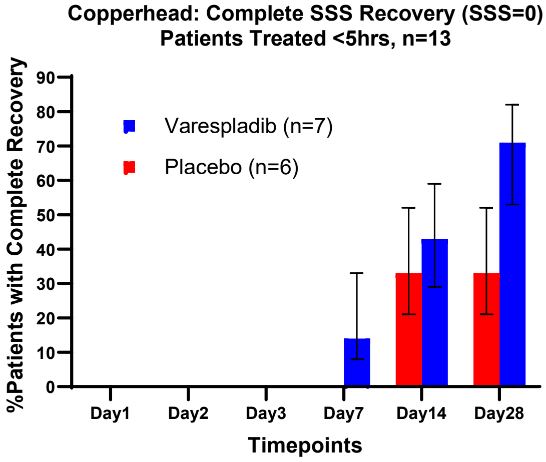

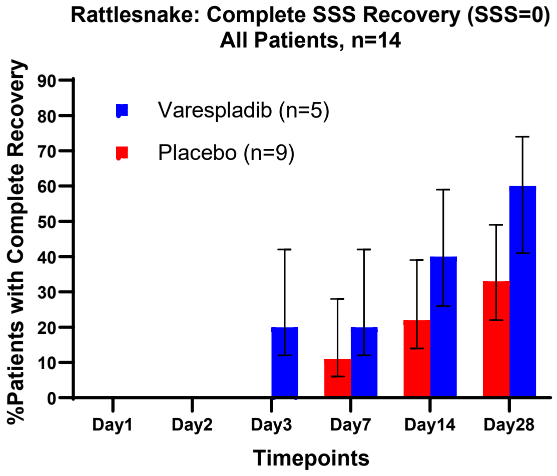

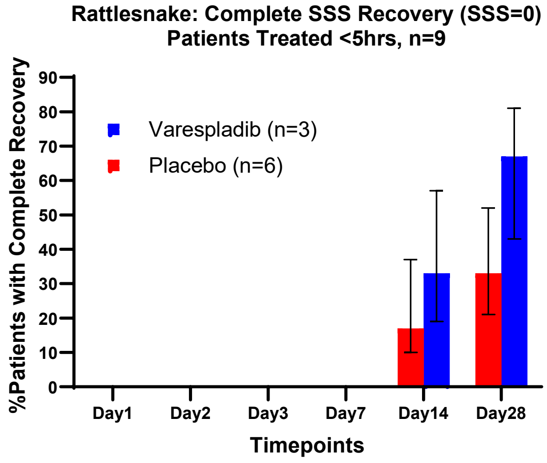

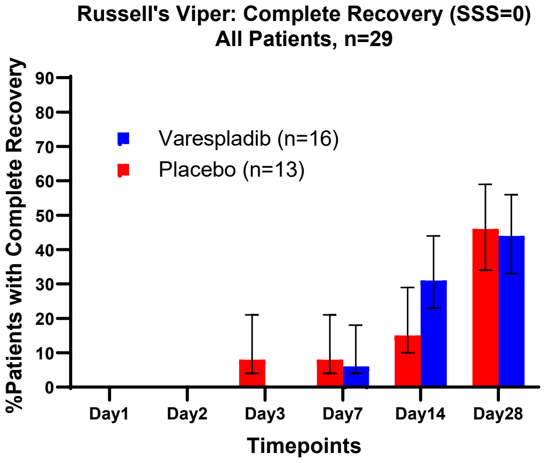

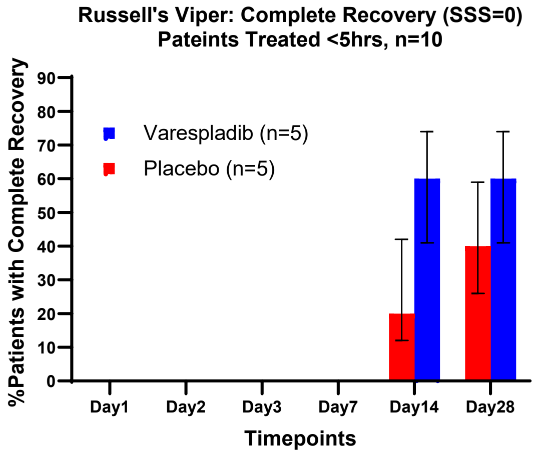

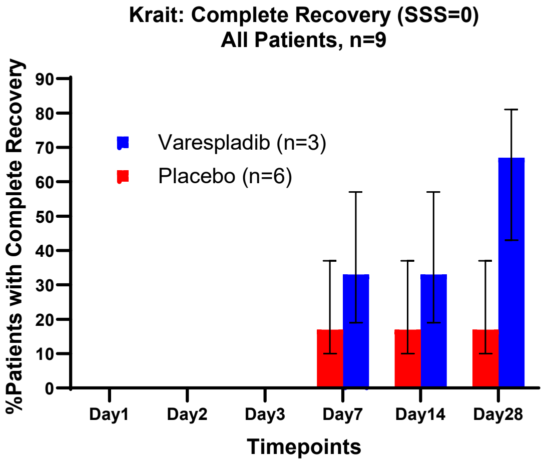

Only two krait bite subjects treated <5hrs. Both subjects were randomized to the placebo group.

Appendix 7: Snakebite Severity Score subscores, by treatment group, for all patients and for the subgroup of patients treated within 5 hours. Subscores only shown for patients with non-zero subscores at baseline. Error bars are standard errors.

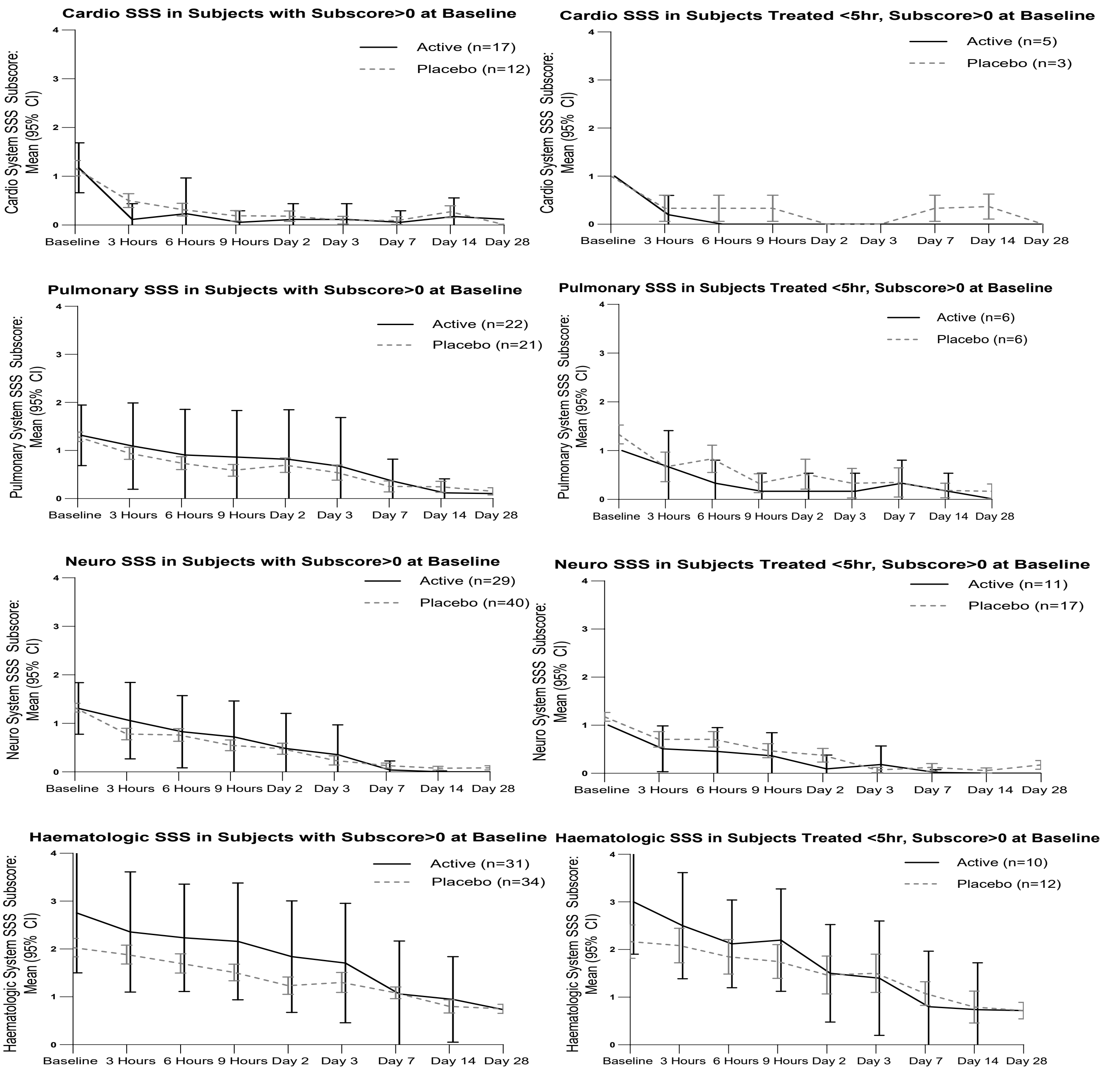

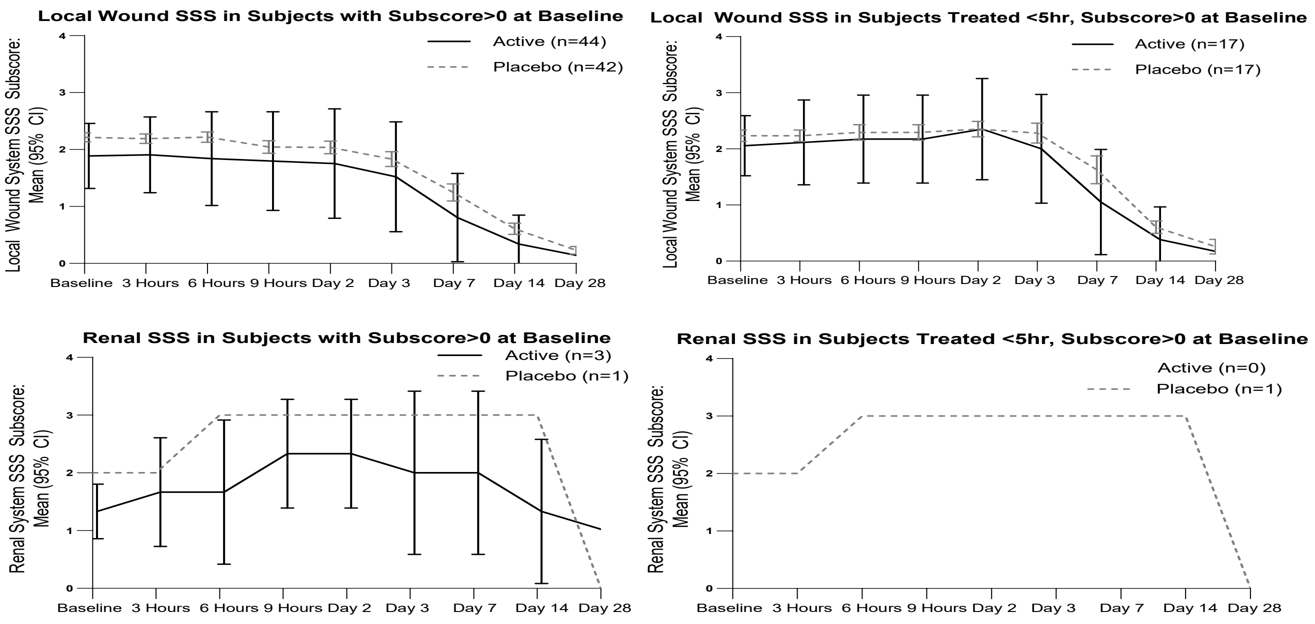

Appendix 8: Primary outcome, change in SSS from baseline to average at 6 and 9 hours, by pre-specified subgroups in the intention-to-treat population (n=95).

Outcome	Varespladib		Placebo		Treatment Effect* (95% CI)
	N	Mean (SE)	N	Mean (SE)	
All patients					
Change in SSS from baseline to average at 6 and 9 hours	45	1.1 (0.3)	50	1.5 (0.2)	-0.4 (-0.8 to 0.1) (Favors placebo)
Subgroups					
Age group					
5 to 17 years	6	0.9 (0.3)	4	1.3 (0.4)	-0.4 (-1.1 to 0.2)
18 and older	39	1.1 (0.2)	46	1.4 (0.2)	-0.3 (-0.8 to 0.2)
Antivenom completed prior to baseline					
Yes	19	0.6 (0.3)	16	1.0 (0.3)	-0.5 (-1.0 to 0.1)
No	26	1.8 (0.4)	34	2.0 (0.4)	-0.2 (-0.9 to 0.4)
Country					
India	31	1.1 (0.3)	31	1.4 (0.3)	-0.4 (-1.0 to 0.2)
U.S.	14	1.3 (0.4)	19	1.3 (0.3)	0.0 (-0.5 to 0.4)
Snake Type					
Viper	36	1.1 (0.3)	34	1.4 (0.3)	-0.3 (-0.7 to 0.2)
Elapid	3	-1.2 (1.4)	11	1.4 (0.8)	-2.5 (-5.4 to 0.3)
Unknown	6	2.0 (0.5)	5	2.1 (0.7)	-0.2 (-1.3 to 1.0)
Time from bite to study drug					
< 5 hours	17	1.6 (0.6)	20	1.1 (0.6)	0.5 (-0.3 to 1.2)
≥ 5 hours	28	1.0 (0.3)	29	1.7 (0.3)	-0.7 (-1.3 to -0.1)
Moderate to severe baseline neurotoxicity					
Yes	8	1.9 (1.1)	11	3.2 (1.1)	-1.3 (-3.1 to 0.5)
No	37	1.2 (0.2)	39	1.3 (0.2)	-0.1 (-0.5 to 0.4)

Appendix 9: Safety and tolerability measures, pediatric patients aged 5 to 17 years (n=10).

Safety Outcome - no. (%)	Varespladib (n=6)	Placebo (n=4)
Patients with at least one TEAE	1 (16.7%)	3 (75%)
Gastrointestinal disorders	1 (16.7%)	1 (25%)
Nausea	0	1 (25%)
Vomiting	1 (16.7%)	1 (25%)
Diarrhea	1 (16.7%)	0
Infections	0	1 (25%)
Wound Infections	0	1 (25%)
Allergic Reaction to Immunoglobulin	1 (16.7%)	1 (25%)
Acute kidney injury*	0	0
Hepatic enzyme increase**	0	0
Cardiac ischemia	0	0
Skin and subcutaneous tissue disorders	0	2 (50%)
Nervous system disorder	0	1 (25%)
Headache	0	1 (25%)

Appendix 10: Reflexivity Statement

	Questions
Engagement	1. Has the research team engaged constructively with the reflexivity statement?<br><br>Yes. Ophirex was founded as a Public Benefit Corporation in 2015 with the goal of reducing the global burden of snakebite envenoming. Dr. Matt Lewin, who is the founder of Ophirex, is a member of the World Health Organization's (WHO's) Snakebite Envenoming Working Group and his advocacy was important to WHO's decision to list snakebite envenoming as a neglected tropical disease. The design of the BRAVO trial reflects a close partnership between the Ophirex, India investigators, and U.S. investigators. Decisions regarding authorship reflect contributions made to the development of the idea, the development of the protocol, securing of funding, enrollment into the trial, and drafting of the manuscript. All six lead investigators from India are listed as authors as well as three sub-investigators co-Investigators. One additional author is an Indian physician who is a full-time employee of Ophirex. Decisions regarding authorship have been discussed and agreed upon among these individuals.
Co-development	2. Have the research partners co-developed the research study?<br><br>The design of the BRAVO trial reflects input from both India and U.S. colleagues. Prior to designing the protocol, Dr. Matthew Lewin spent a month in India observing the management of snakebite envenoming patients to better under the usual treatment and typical clinical course. One product of this engagement is a manuscript that reflects input from Dr. Ashish Bhalla, a renowned India physician and snakebite envenoming expert, which contemplates outcomes used in the BRAVO trial (see reference below).

	Additionally, Ophirex hired Dr. Stephen Samuel, an Indian physician to provide input on the design and implementation of the trial. Finally, and most importantly, investigators in India provided substantive input on early versions of the protocol and the final design of the study reflects this input. Examples of this include the use of the 20 WBCT, a common bedside test for coagulopathy in India, as an inclusion criterion, and the addition of a new renal subscore as part of the 1st key secondary outcome. The submitted manuscript is the product of a close collaboration between Ophirex, India investigators, and U.S investigators, with all authors making substantive contributions.<br><br>Bickler, P.E., Abouyannis, M., Bhalla, A. and Lewin, M.R., 2023. Neuromuscular weakness and paralysis produced by snakebite envenoming: mechanisms and proposed standards for clinical assessment. *Toxins*, *15*(1), p.49.<br><br>3. Does the study address priority research questions for the LMIC partner(s)?<br><br>Yes. Snakebite envenoming is a major public health problem in India, causing an estimated 50,000 deaths each year. The BRAVO trial tests a novel therapy for snakebite envenoming with the potential for substantive benefit for people in India who are bitten by snakes. The inclusion of 62 patients from India speaks to the scope of the problem in India, the enthusiasm of the India-based investigators, and the sponsor's desire to ensure that the results are relevant for people living in India.
Authorship	4. Is there a LMIC partner who is the first or last author? If not, what is the explanation?<br><br>The BRAVO trial was a complex trial that required input from many different individuals. The selection and order of authors reflects our best effort to recognize the contributions of people to the trial and the preparation of the manuscript. All investigators from India are included as authors.<br><br>5. How have LMIC early career researchers been incorporated as authors?<br><br>Junior investigators at sites in both the U.S. and India are included as authors. Specifically, in India this includes Dr. Harish Kumar, Dr. Madhu Ravikumar, and Dr. Sujoy Sarkar.
Dissemination	6. How are data shared with LMIC partners to address research needs?<br><br>The data from the clinical trial are available to all investigators in the trial, including all India investigators. In preparation of the submitted manuscript, multiple drafts of the manuscript and the associated analyses have been shared with colleagues in India as well as the United States. Their input has informed the submitted work. Additionally analyses and manuscripts resulting from the trial are being prepared in close collaboration between Indian and U.S. investigators.<br><br>7. Is there open access funding to improve publication dissemination?<br><br>Yes. Thank you for making this option possible though BMJ Global Health. Ophirex is a Public Benefit Corporation, and we have an internal policy that all submitted manuscripts will be made Open Access.
